# Molecular identification and transmission studies of X-cell parasites from Atlantic cod *Gadus morhua *(Gadiformes: Gadidae) and the northern black flounder *Pseudopleuronectes obscurus *(Pleuronectiformes: Pleuronectidae)

**DOI:** 10.1186/1756-3305-4-15

**Published:** 2011-02-08

**Authors:** MA Freeman, M Eydal, M Yoshimizu, K Watanabe, AP Shinn, K Miura, K Ogawa

**Affiliations:** 1 Institute of Ocean and Earth Sciences & Institute of Biological Sciences, University of Malaya, Kuala Lumpur, Malaysia; 2 Institute of Aquaculture, University of Stirling, FK9 4LA, Scotland, UK; 3 Institute for Experimental Pathology, University of Iceland, Keldur, Reykjavík, Iceland; 4 Laboratory of Fish Diseases, The University of Tokyo, Tokyo 113-8657 Japan; 5 Laboratory of Microbiology, Hokkaido University, Hakodate 041-8611, Japan; 6 Department of Aquatic Bioscience and Industry Faculty of Bio-Industry, Tokyo University of Agriculture, Abashiri, Hokkaido 099-2493, Japan; 7 Hokkaido Central Fisheries Experiment Station, Yoichi 046-8555, Japan

## Abstract

**Background:**

Epidermal pseudotumours from *Hippoglossoides dubius *and *Acanthogobius flavimanus *in Japan and gill lesions in *Limanda limanda *from the UK have been shown to be caused by phylogenetically related protozoan parasites, known collectively as X-cells. However, the phylogenetic position of the X-cell group is not well supported within any of the existing protozoan phyla and they are currently thought to be members of the Alveolata.

Ultrastructural features of X-cells in fish pseudotumours are somewhat limited and no typical environmental stages, such as spores or flagellated cells, have been observed. The life cycles for these parasites have not been demonstrated and it remains unknown how transmission to a new host occurs.

In the present study, pseudobranchial pseudotumours from Atlantic cod, *Gadus morhua*, in Iceland and epidermal pseudotumours from the northern black flounder, *Pseudopleuronectes obscurus*, in Japan were used in experimental transmission studies to establish whether direct transmission of the parasite is achievable. In addition, X-cells from Atlantic cod were sequenced to confirm whether they are phylogenetically related to other X-cells and epidermal pseudotumours from the northern black flounder were analysed to establish whether the same parasite is responsible for infecting different flatfish species in Japan.

**Results:**

Phylogenetic analyses of small subunit ribosomal DNA (SSU rDNA) sequence data from Atlantic cod X-cells show that they are a related parasite that occupies a basal position to the clade containing other X-cell parasites. The X-cell parasite causing epidermal pseudotumours in *P. obscurus *is the same parasite that causes pseudotumours in *H. dubius*. Direct, fish to fish, transmission of the X-cell parasites used in this study, via oral feeding or injection, was not achieved. Non-amoeboid X-cells are contained within discrete sac-like structures that are loosely attached to epidermal pseudotumours in flatfish; these X-cells are able to tolerate exposure to seawater.

A sensitive nested PCR assay was developed for the sub clinical detection of both parasites and to assist in future life cycle studies. PCR revealed that the parasite in *P. obscurus *was detectable in non-pseudotumourous areas of fish that had pseudotumours present in other areas of the body.

**Conclusions:**

The inability to successfully transmit both parasites in this study suggests that either host detachment combined with a period of independent development or an alternate host is required to complete the life cycle for X-cell parasites. Phylogenetic analyses of SSU rDNA confirm a monophyletic grouping for all sequenced X-cell parasites, but do not robustly support their placement within any established protist phylum. Analysis of SSU rDNA from X-cells in Japanese flatfish reveals that the same parasite can infect more than one species of fish.

## Background

X-cell disease in fish typically develops either as epidermal pseudotumours, gill filament lesions or pseudobranchial swellings in various marine species [1]. X-cells associated with epidermal pseudotumours in the flathead flounder, *Hippoglossoides dubius *Schmidt, 1904 and the yellowfin goby *Acanthogobius flavimanus *(Temminck et Schlegel, 1845) from northern Japan, have been shown, using small subunit ribosomal DNA (SSU rDNA) sequence data, to be related protozoan parasites that have an unresolved taxonomic identity [2]. Freeman [1] further demonstrated that the X-cell parasite causing gill filament lesions in the European dab, *Limanda limanda *(L., 1758), is related to the two Japanese X-cell parasites, and suggested they belong in the alveolate group and that they are basal members of the Myzozoa. Pseudobranchial X-cell pseudotumours occur in gadoid fish from the Pacific and Atlantic Oceans [3], but thus far have not been studied phylogenetically.

In the coastal waters of Hokkaido, seven species of pleuronectid flatfish have been reported to have epidermal pseudotumours containing X-cells [4]. Of these seven species, only X-cells from *H. dubius *have been characterised using SSU rDNA analyses [5], and it is not known how host specific X-cell parasites are, and whether the same X-cell parasite is responsible for causing epidermal pseudotumours in more than one flatfish species.

Experimental transmission of X-cell disease between fish has been attempted, but has never convincingly been achieved. However, most transmission studies were based on the assumption, at the time, that the X-cell condition had a viral aetiology and some studies may not have been suitable for the successful experimental transmission of protozoan parasites. A cell-free homogenate of epidermal pseudotumour tissue from the yellowfin goby, *A. flavimanus*, was subcutaneously inoculated into uninfected individuals, but no pseudotumour growth was observed during the trial [6]. Gill lesion regression was observed in European dab, *L. limanda*, that were being maintained in the laboratory, and subsequent attempts to transmit the X-cell condition to uninfected fish using an inoculum derived from X-cell material were not successful [7]. Cohabitation experiments with Atlantic cod, *Gadus morhua *L. 1758, were conducted by Morrison *et al*. [3], but were inconclusive due to the high mortalities of wild-caught X-cell infected fish under experimental conditions and the uncertainty that visibly uninfected wild-caught cod were truly naïve at the start of the experiment. However, a single uninfected fish did develop a large unilateral lesion after two months cohabitation with an X-cell infected cod.

In the present study we sampled pseudobranchial pseudotumours from Atlantic cod, *G. morhua*, from Iceland and epidermal pseudotumours from the northern black flounder, *Pseudopleuronectes obscurus*, (Herzenstein, 1890) from northern Japan. Experimental transmissions of X-cell parasites from Atlantic cod and northern black flounder to naïve fish were attempted and a sensitive PCR assay was developed for their sub-clinical detection. In addition, SSU rDNA analyses were utilised in order to confirm whether the same parasite is responsible for infecting different flatfish species in northern Japan, and whether the X-cell parasite causing pseudobranchial pseudotumours in Atlantic cod is phylogenetically related to those causing epidermal tumours and gill filament lesions in flatfish and gobies.

## Methods

### Sampling and transmission studies of X-cell parasites from cod in Iceland

Pseudobranchial pseudotumours were excised from naturally infected young Atlantic cod selected from a land based cod farm in North West Iceland that is stocked with a local population of wild juvenile cod caught in an adjacent fjord for on-rearing. A sub-sample of pseudotumour tissue from each fish was fixed in 10% buffered formalin and 95% ethanol for histology and DNA analyses respectively; the remainder was briefly stored at 4°C until required. Pseudobranchial tissues from cod with no pseudobranchial swellings were fixed in 95% ethanol for use as negative control tissue in the DNA study. The pseudotumour tissue was gently homogenised in PBS with a sterile pestle and mortar and diluted with PBS to allow sufficient homogenate for the experiment. The homogenate was viewed under a compound microscope to ensure that intact X-cells were present. Disease free hatchery-reared juvenile cod, used in the experiment, came from the hatchery station of the Marine Research Institute in Iceland at Staður, Grindavík and the trial was conducted at the Sandgerði Marine Centre, Iceland. Fish were maintained in 1.5 m diameter rearing tanks and supplied with clean bore-hole sea water at a constant temperature of 9°C.

Experimental fish were split into three groups and kept in separate tanks. One group was orally intubated (oral group, n = 13) with 1 ml of pseudotumour homogenate using a flexible 3 ml plastic pipette; a second group was given an intracoelomic (IC) injection (IC group, n = 15) of 100 μl of pseudotumour homogenate; and a control group (n = 10) received no treatment. The fish were observed and fed daily and the trial ran for 17 weeks. At the start of trial fish had a mean weight of 65 g and a mean length of 19 cm.

Three fish from each group were sacrificed and examined for signs of pseudotumour development nine weeks into the trial, and the remaining fish were examined on week 17 at the end of the trial (oral group, n = 10; IC group, n = 4; control group, n = 6). All pseudobranchial tissues were removed, longitudinally bisected and fixed in either 95% ethanol for DNA analyses or 10% buffered formalin for histology. Nine fish that died during the trial (weeks 1-9) were frozen until week 17 and examined with those that survived the duration of the experiment.

### Sampling and transmission studies of X-cell parasites from flatfish in Japan

*Pseudopleuronectes obscurus *with typical X-cell epidermal pseudotumours were taken as a by-catch by shrimp fishermen during the summer shrimp fishing season in Notsuke bay in Eastern Hokkaido, Japan. Infected fish were maintained in tanks until required for the experimental infections. Pseudotumours were dissected from the underlying tissues in recently culled fish. Some pseudotumour tissue was fixed in 10% buffered formalin and 95% ethanol for histology and DNA analyses respectively, the remainder was minced with scissors, half being set aside for the feeding experiment and the rest gently homogenised in physiological saline using a pestle and mortar and a ground-glass tissue homogeniser. The resulting homogenate was passed through a fine gauze cloth to remove large pieces of connective tissue and centrifuged at 1800 g for 5 min. The pellet was resuspended in 3 ml of saline, examined microscopically for the presence of intact X-cells and used immediately as an inoculum in the fin base injection experiment. Negative control skin samples, for the DNA study, were also taken from uninfected areas of X-cell infected fish and from fish with no visible signs of pseudotumours.

X-cells from discrete sac-like structures, found at the extremities of epidermal pseudotumours, were also examined microscopically and placed in seawater in 24-well tissue culture plates and maintained at 15°C to observe any amoeboid forms and monitor their ability to adhere to plastic over a 24 hour period.

Fish used in the transmission experiments were hatchery reared and assumed to be free from disease. Two species of flatfish were used in the experimental infections: barfin flounder, *Verasper moseri *(Jordan et Gilbert, 1898), family Pleuronectidae (righteye flounders); and Japanese flounder, *Paralichthys olivaceus *(Temminck et Schlegel, 1846), a lefteye flounder, family Paralichthyidae (Large-tooth flounders). In total, 150 fish of each species were used; fifty as control fish, fifty for the feeding of infected material and fifty for the fin base injection experiment.

For the feeding experiment, small pieces of pseudotumour tissue approximately 1 mm^3 ^in size were fed directly to fish that had been starved for two days and their feeding behaviour was observed to ensure that fish fed uniformly. For the subcutaneous fin base injection experiment, fish were anaesthetised and 25 μl of inoculum injected with a fine needle at the base of the dorsal fin rays. 25 μl was considered the largest volume to safely inject into fish smaller than 50 mm total length. Control fish received no treatment. Experimental fish were kept in six separate tanks in a flow-through system at 15°C in full strength seawater, and observed for three months. Total length measurements of fish at the beginning of the experiment were: barfin flounder 35-49 mm (mean 41.9 mm; n = 20) and Japanese flounder 23-37 mm (mean 30.8 mm; n = 20). At the end of the experiment, fish were examined using a dissecting microscope for the presence of epithelial pseudotumours on the skin and fins. PCR was not performed on tissue samples from the fin base injection sites for the flatfish group.

### Histological examination of pseudotumours

Fresh pseudotumours were fixed in 10% buffered formalin for 48 h and transferred to 70% ethanol for processing in an automatic tissue processor. After embedding in wax, blocks were trimmed and sections of 5 μm were cut on a Reichert-Jung Biocut microtome before being stretched on a water-bath at 45°C and floated on to slides. Slides were dried overnight in an oven at 60°C prior to staining with haematoxylin and eosin. The sac-like structures from *P. obscurus *were carefully removed, from live fish, using forceps, and fixed in 2.5% glutaraldehyde in 0.1 M Sorensen's phosphate buffer (pH 7.4) at 4°C for 4 h. Fixed tissues were then rinsed in 0.1 M Sorensen's phosphate buffer (pH 7.4) at 4°C overnight before being post-fixed in 1% osmium tetroxide for 1 h, dehydrated through an ethanol series, embedded in Spurr's resin and polymerised at 60°C for 48 h. Semi-thin sections of 0.5 μm thickness were cut using a glass knife on a Reichert Ultracut E ultramicrotome and stained with 1% Azur II followed by 1% methylene blue in 1% borax (50:50).

### DNA extraction, PCR amplification and SSU rDNA sequencing of X-cell parasites

Ethanol fixed pseudotumour material and negative control tissues were homogenised with a sterile Eppendorf pestle and digested overnight at 56°C in 0.4 ml high concentration urea buffer containing 100 μg/ml proteinase K. DNA was extracted using a QIAamp DNA Mini Kit (QIAGEN Inc.) following the manufacturer's tissue protocol and used as template DNA for subsequent PCR reactions.

Partial SSU rDNA for both X-cell parasites was first amplified using the primer pair: H-F1 M 5' gttctttcttgattctatrag 3' and H-R3 M 5' taggaattcctcgttcaagacg 3' that were modified from degenerate haplosporidian primers [8], and require a 48°C annealing temperature. Universal primers 18e [9] and 606f/r and 18gM [10] were used in combination with the above primers and with more specific X-cell primers designed from initial sequence reads (Table 1). All PCRs were performed in 20 μl volumes containing ~10 ng of genomic DNA, 15 pmol of each primer, 0.25 mM of dNTP, PCR buffer with a final MgCl_2 _concentration of 2 mM and 0.5 units of Taq DNA polymerase. After an initial denaturation at 95°C for 5 min, samples were subjected to 35 cycles of amplification (denaturation at 95°C for 30 s, primer annealing at 55°C for 30 s (unless otherwise stated), and extension at 72°C for 1 min), followed by a 7 min terminal extension at 72°C. PCR amplicons were purified using a PCR purification kit (QIAGEN Inc) and used directly in sequencing reactions. The sequencing reactions were performed using BigDye^® ^Terminator Cycle Sequencing chemistry utilising the same oligonucleotide primers that were used for the PCRs. DNA sequencing was completed on amplicons from four infected fish for each species. Sense and anti-sense strands were sequenced for all PCR products and contiguous sequences constructed manually using CLUSTAL_X [11] and BioEdit [12]. CLUSTAL_X was used for the sequence alignments with the settings for gap opening/extension penalties being adjusted manually to achieve optimum alignments. Regions of ambiguous sequence alignments were manually edited using the BioEdit sequence alignment editor and final alignments of specific taxa were generated using CLUSTAL_X.

**Table 1:** PCR primers used for the nested amplification of X-cell SSU rDNA.

nested primer pairs	fwd primer (5'-3')	rev primer (5'-3')	annealing temp °C	size (bp)
cod				
i) 305f^s^/H-R3M^x^	tgacctatcatgctgtgatgg	taggaattcctcgttcaagacg	54	1350
ii) 390Xf^x^/1400r^s^	agagggagcctgagagacg	agcaagcccgtatggagaagacg	52	1000
flounder				
i) 280f^s^/H-R3M^x^	atccatcagccatcgacgc	taggaattcctcgttcaagacg	55	1290
ii) 390Xf^x^/1000r^s^	agagggagcctgagagacg	tcgtccgatcctcagtcgg	55	645

^s ^X-cell species-specific primer

^x ^X-cell universal primer

Appropriate taxa were chosen for the phylogenetic analyses by performing nucleotide BLAST searches with the X-cell sequences [13] and reviewing previous phylogenetic analyses that included X-cell sequence data [1,2,5]. Additional taxa were chosen to further represent the major protist phyla (see Additional file 1).

Phylogenetic analyses were conducted using maximum parsimony (MP) methodologies in PAUP*4.0 beta10 [14]. MP analysis was done using a heuristic search with tree bisection-reconnection (TBR) branch swapping, 10 random taxon addition replicates, using the accelerated transformation (ACCTRAN) option. Gaps were treated as missing data and clade support was assessed using bootstrapping with 1000 replicates. Bayesian inference (BI) analyses were conducted using MrBayes v. 3.0 [15]. Models of nucleotide substitution were evaluated for the data using MrModeltest v. 2.2 [16]. The most parameter-rich evolutionary model based on the AIC was the general time-reversible, GTR+I+G model of evolution. Therefore, the settings used for the analysis were nst = 6, with the gamma-distributed rate variation across sites and a proportion of invariable sites (rates = invgamma). The priors on state frequency were left at the default setting (Prset statefreqpr = dirichlet (1,1,1,1)). Posterior probability distributions were generated using the Markov Chain Monte Carlo (MCMC) method with four chains being run simultaneously for 1,000,000 generations. Burn in was set at 2500 and trees were sampled every 100 generations making a total of 7500 trees used to compile the consensus trees.

### Specific nested PCR detection system

A highly sensitive and specific nested PCR detection assay was developed for the X-cell parasites from Atlantic cod and the northern black flounder, using the newly acquired SSU rDNA sequence data. Oligonucleotide primers were designed that were universal for all currently known X-cell sequences and specific primers were also designed for each new X-cell parasite sequence. These primers were then used in the (universal/specific) combinations described in Table 1. Nested PCR reactions were conducted using the same PCR conditions described above, but with adjusted annealing temperatures (Table 1), the products of the first round reaction were used as template DNA for the second reaction.

In accordance with section 8.6 of the ICZN's International Code of Zoological Nomenclature, copies of this article are deposited at the following five publicly accessible libraries: Natural History Museum, London, UK; American Museum of Natural History, New York, USA; Museum National d'Histoire Naturelle, Paris,

France; Russian Academy of Sciences, Moscow, Russia; Academia Sinica, Taipei, Taiwan.

## Results

**Figure 1:**
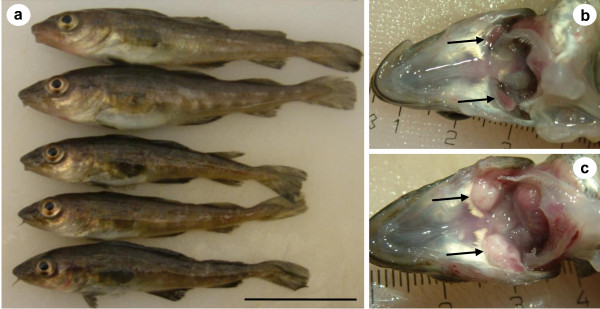
**Pseudobranchial pseudotumours in Atlantic cod *Gadus morhua *from Iceland**. a) Five cod from the same year-class; two uninfected fish (top) and three infected fish (bottom). b) Pseudobranchs have a gill-red colour in healthy fish and are enlarged and have a creamy-whitish appearance when infected (c) (black arrows). Scale bars a = 4 cm, b & c visible.

Atlantic cod with pseudobranchial pseudotumours were hand-selected from schooling juvenile fish in large indoor tanks. Infected cod appeared to be darker in colour and generally smaller than uninfected ones from the same year class (Figure 1a). Infected pseudobranchs were significantly enlarged and had a creamy-whitish appearance, whereas uninfected pseudobranchs had a normal gill-red colour (Figure 1b &1c). The excised pseudobranchial pseudotumours were approximately 0.8 cm in length from fish that measured 10-12 cm in total length. The pseudobranchial pseudotumours from six bilaterally infected fish were used in the transmission experiment.

**Figure 2:**
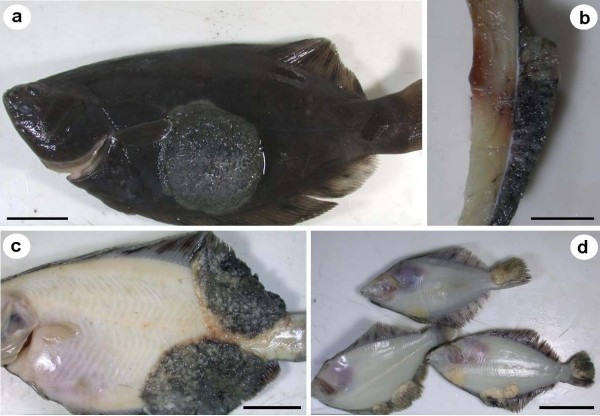
**Epidermal X-cell pseudotumours on formalin-fixed *Pseudopleuronectes obscurus *from Hokkaido, Japan**. a) A large central dorsal pseudotumour, seen in cross section (b). c) Pseudotumours can extend from the dorsal surface to the ventral surface and remain pigmented. d) Juvenile fish are also infected and ventral pseudotumours can also be unpigmented. Scale bars a, c & d = 3 cm, b = 10 mm.

**Figure 3:**
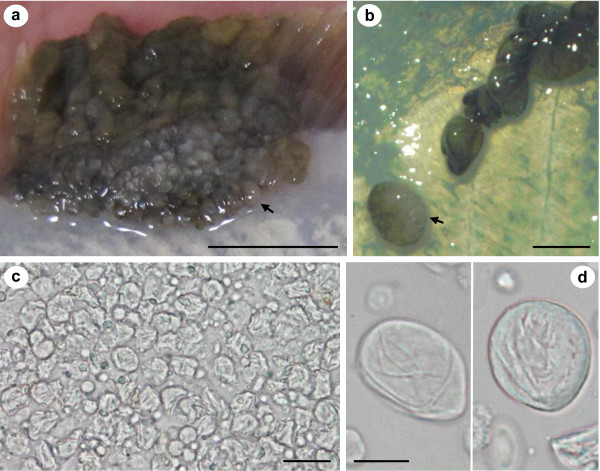
**Sac-like structures at the extremities of an epidermal pseudotumour on a live specimen of *Pseudopleuronectes obscurus *and X-cells released from the sacs in tissue culture plates**. a & b) Appearance of a fresh epidermal pseudotumour showing sac-like structures that can be separated from the main part of the pseudotumour intact, black arrows indicate the same sac attached and detached in a & b respectively. c) X-cells and host tissue debris immediately after being released from a sac-like structure. d) X-cells maintained in plastic tissue culture plates containing seawater, 24 hrs after being released from a sac-like structure. Scale bars a = 10 mm, b = 2 mm, c = 20 μm, d = 10 μm.

*Pseudopleuronectes obscurus *with epithelial pseudotumours caused by X-cell infection were caught in Notsuke Bay, Hokkaido (Figure 2a-d). Epithelial pseudotumours were typical of those described from numerous flatfish species from Hokkaido and were often large and significantly raised from the body surface, but did not penetrate the underlying musculature (Figure 2a &2b), making them easy to remove from the fish to prepare the homogenate for the transmission experiment. Pseudotumours were always pigmented on the ocular (eyed) side, but could be either pigmented or non-pigmented on the abocular (non-eyed) side (Figure 2c &2d) and when sampled, juvenile fish were also sometimes infected (Figure 2d). Pseudotumours often extended to the edge of the fins (Figure 2c & Figure 3a). Here, and at other margins of the pseudotumour, sac-like structures were observed that could be detached, intact, with mild pressure (Figure 3b). The sacs were relatively fragile, easy to rupture and contained X-cells and other cellular debris (Figure 3c). X-cells from ruptured sacs that had been maintained in seawater for 24 hrs showed no signs of adhering to the plastic tissue culture plates, had no pseudopodia and were not amoeboid in form. After 24 hrs in seawater they appeared similar to ones that were freshly removed from sacs and had not suffered any noticeable shrinkage (Figure 3d).

**Figure 4:**
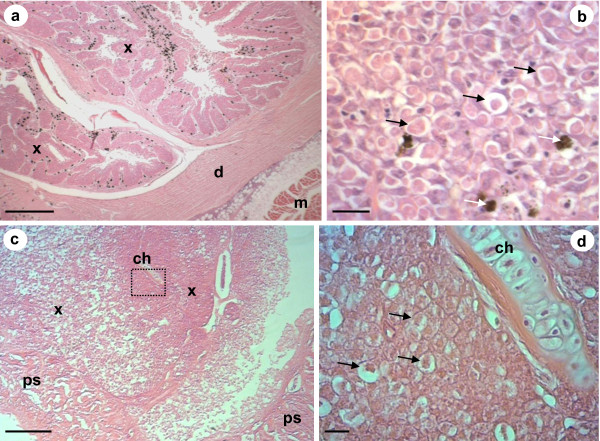
**Haematoxylin and eosin stained histological sections of X-cell pseudotumours**. a) Low magnification of a section through an epidermal pseudotumour in *P. obscurus*. Large numbers of X-cells (x) are in folds of infected host tissues. The dermis (d) and the underlying muscle (m) remain uninfected. b) High power magnification of X-cells in *P. obscurus *have a characteristic polygonal appearance with a lightly staining large nucleus (black arrows), melanomacrophages are present in high numbers (white arrows). c) Low magnification of a section through a pseudobranchial pseudotumour in juvenile Icelandic cod. X-cell masses (x) form adjacent to pseudobranchial tissues (ps) but are also found surrounding pseudobranchial cartilage containing chondrocytes (ch) deeper into the pseudotumour. d) High power magnification of the boxed section in Figure 3c; numerous X-cells (black arrows) form a distinctive mass that forms the bulk of the pseudotumour, pseudobranchial cartilage containing chondrocytes (ch) is enclosed by X-cells. Scale bars a & c = 400 μm, b & d = 20 μm.

Histological analyses of the pseudotumours of cod and northern black flounder revealed that X-cells were present in large numbers in both pseudotumour types (Figure 4a-d). In *P. obscurus*, the X-cells were limited to the epidermal skin layer and were arranged in a regular pattern of folded tissues that contained numerous melanomacrophages and other unidentifiable host cells (Figure 4a &4b). In cod the X-cells appeared to be adjacent to, but not intermingled with, pseudobranchial tissue (Figure 4c). However, pseudobranchial cartilages were also seen surrounded by X-cells deeper into the pseudotumour lesions (Figure 4d).

**Figure 5:**
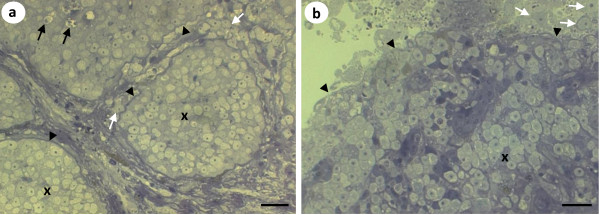
**Semi-thin sections of the sac-like structures from *Pseudopleuronectes obscurus***. a) In the central region of the sac, X-cells (x) are found to be mostly restricted to large masses within the host epidermal tissue contained by a thick basal lamina-like membrane (black arrowheads), under which lies connective tissue. Some X-cells inside the large aggregations appear to be degenerate (black arrows), whilst others can be found amongst the connective tissues (white arrows) outside of the membrane that contains the majority of the X-cells. b) At the border of the sac there is a less organised tissue structure observed. Aggregations of X-cells (x) are no longer contained within membranous structures and numerous X-cells are present at the peripheral margin of the sac (black arrowheads) and some appear to be independent from the sac (white arrows). Scale bars 20 μm.

Semi-thin sections of the sac-like structures show that in the central region of the sac, X-cells are found to be mostly restricted to large masses within the host epidermal tissue contained by a thick basal lamina-like membrane with underlying connective tissues. Some X-cells inside these large masses appear to be degenerate, whilst others were observed amongst the connective tissues outside of the membrane that contains the majority of the parasites (Figure 5a). At the border of the sac there is a less organised tissue structure observed. Aggregations of X-cells are no longer contained within membranous structures and they are less densely packed together with numerous X-cells being present at the peripheral margin of the sac and some appearing to be external to the sac (Figure 5b).

Visual inspection of cod pseudobranchial tissues and microscopic inspection of both flatfish species at the end of the transmission experiments failed to detect the presence of abnormal tissue growth or signs of pseudotumours developing. Furthermore, PCR analyses of pseudobranchial DNA from experimental cod did not detect the presence of cod X-cell parasite DNA. PCR was not performed for the infection trials of the flatfish group.

Nested PCRs were successfully developed for both X-cell parasite species (Table 1), and no cross reactivity occurred between the two types of X-cells during PCR analyses. During the development of the nested PCR assay, negative control tissue from infected *P. obscurus *(skin samples taken >2 cm from pseudotumour sites), were sometimes found to be positive for the presence of X-cell DNA in the second round of the PCR; whereas skin samples from uninfected *P. obscurus *remained negative during both rounds of the PCR. Negative control tissues from visibly uninfected cod were not found to contain X-cell DNA with the nested PCR.

Partial SSU rDNA sequence data from the X-cell parasites infecting cod, *G. morhua*, and the northern black flounder, *P. obscurus*, were successfully obtained and have been deposited in GenBank (*G. morhua *X-cell, 1728 bp GU296508; *P. obscurus *X-cell, 1712 bp GU296509). A BLAST search with the contiguous X-cell sequence from *P. obscurus *showed a very high homology (> 99%) with the X-cell sequence reported from *H. dubius *[5]. A more detailed comparison with the X-cell sequence from *H. dubius *revealed a 99.76% similarity over 1692 bases of comparable data, confirming that the two isolates from different flatfish hosts should be considered conspecific.

**Figure 6:**
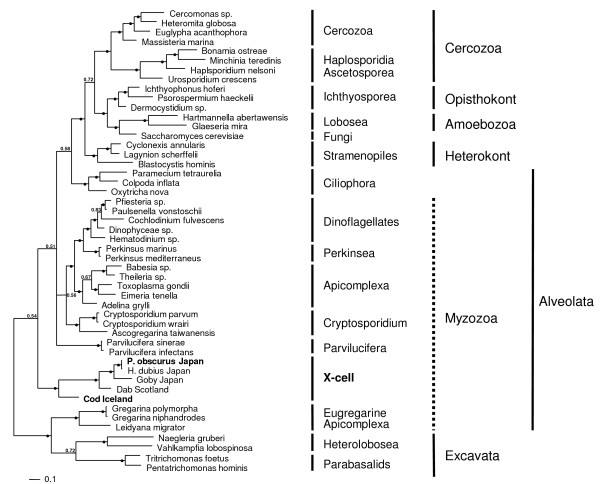
**Bayesian Inference phylogenetic tree**. Phylogenetic tree constructed using Bayesian Inference analyses using the general time-reversible (GTR+I+G) model of evolution. The numbers at the nodes represent the clade posterior probability, indicating the proportion of sampled trees that contained that branch. A solid circle at the node indicates a posterior probability of 0.9 and above. To the right of the tree are the currently accepted taxonomic rankings (order, class, phylum), at the far right are the higher scientific classifications (super-phylum).

Maximum parsimony and Bayesian phylogenetic analyses produced trees with a similar topology. The Bayesian inference tree (Figure 6) shows that all known X-cell sequences group together to form a monophyletic clade that is within the alveolates and is basal to the majority of the myzozoan taxa. The sequence from *P. obscurus *groups with the sequence from *H. dubius *and collectively they form a clade with the species infecting the yellowfin goby from Japan. More basal to the Japanese/Pacific grouping is the X-cell parasite from European dab and basal to this main X-cell group is the sequence for the Atlantic cod pseudobranchial parasite. This monophyletic grouping for the X-cell parasites was robustly supported with internal probabilities of 1.0 at all nodes within the group. However, it was less well supported from the main spine of the tree with a probability of 0.54 and did not group with other protist phyla.

## Discussion

The inability to successfully transmit both X-cell parasites in this study indicates that either the experimental design or conditions were not suitable for transmission to take place or that the X-cell stages seen in fish pseudotumours are not infective to new fish hosts. As comprehensive precautions, described in the methods, were taken in preparation for and throughout the transmission trials, we conclude that fish to fish transmission of X-cells is not readily achievable using feeding of infected tissues or by injection of an X-cell inoculum. Ideally we would have been able to use more fish and run the experiments for a longer period. However, the availability of infected tissues for feeding and homogenate preparations was limited.

We have shown that non-flagellate and non-amoeboid X-cells are easily shed from mature epidermal pseudotumours in discrete sac-like structures. These X-cells appear to lack a suitable apparatus with which to successfully infect a new fish host, unless direct transmission is via ingestion of the sacs. However, if simple ingestion of sloughed off sacs were sufficient then feeding of freshly minced tumour tissue should have resulted in transmission of the parasite. Therefore, we believe that it is more likely that the X-cells undergo further development in the environment or in an intermediate host before reinfection of the fish host can occur. The continued support for X-cells being phylogenetically related to the Myzozoa would also indicate that a flagellated life stage is present at some point in the life cycle which would be more capable of finding and infecting a new host fish.

### Amoeboid X-cell forms

As no environmental spore-like or flagellated stages have been described from X-cell pseudotumours in fish, in spite of numerous thorough histopathological and ultastructural studies [1], it is not immediately obvious how transmission to a new host might take place naturally. However, there have been several descriptions of amoeboid-like forms in advanced and ruptured X-cell pseudotumours which may represent the infective agent for a new host or a free-living stage leaving the fish host. Small amoeboid forms of X-cells have been described at the boundaries of epidermal pseudotumours in flatfish from Japan [17], free-floating amoeboid X-cells have been observed in advanced pseudobranchial pseudotumours in Atlantic cod [18] and free X-cells seen in the interlamellar spaces of ruptured gill lesions in the American plaice *H. platessoides *[19]. Freeman [1] recently suggested that transmission of X-cells from the fish host could be via these amoeboid stages and that this amoeboid phase to their life cycle would most likely be present in marine sediments and indeed most fish species known to become infected with X-cells are closely associated with the benthic environment [1]. However, recent molecular phylogenetic studies [[1,2,5], present study] do not support the hypothesis that X-cells are amphizoic amoebae having a free-living stage in coastal marine sediments. Furthermore, in the present study we have clearly demonstrated that the X-cells contained in sac-like structures are not amoeboid in form, do not adhere to plastic culture plates and do not form pseudopodia. We consider this sac stage to be the most likely form to naturally detach from the host fish as they are delicately attached to the main pseudotumour and they are seemingly a discrete unit. During the histological analysis of these structures, the margins of the sacs were not found to contain X-cells in the typical organised pattern that is observed in other parts of the pseudotumour. Rather, these X-cells are less densely packed together and some appear outside the sac membrane. This could signify that the sacs ultimately degenerate and release the X-cells into the environment. In addition, X-cells from the sacs appear to be able to withstand full salinity seawater, indicating that they are capable of surviving in the marine environment.

During the development of the diagnostic PCR, visibly uninfected areas of skin from infected *P. obscurus *were found to contain X-cell DNA. This supports the findings by Yamazaki et al. [17] who described the presence of very small amoeboid-like 'wandering' X-cells, only 3-4 μm in diameter, at the boundaries of epidermal pseudotumours in flatfish from northern Japan. These data suggest that amoeboid forms of X-cells do exist in fish tissues and are probably responsible for autoinfection within the host, causing new pseudotumour growth once host invasion and initial development has occurred.

### Experimental design

It is possible that the methods used in the present study were not suitable for parasite transmission to occur between fish. Nevertheless, the transmission of fish parasites with monoxenous life cycles between fish using similar methods to the ones used in this study has been achieved for various fish parasites [20,21]. However, successful parasite transmission can also be affected by environmental conditions such as salinity and temperature. Therefore, in order to reduce the likelihood of this occurring, in our experiments we endeavoured to use seawater whose temperature and salinity were the same as those of the seawater of the natural environment from where infected fish were taken.

If the release of X-cells from mature or ruptured pseudotumours is required for transmission to a new host, and transmission between fish is possible without an alternate host, then long-term cohabitation experiments in tanks with suitable substrate cover, allowing time for the development of an environmental stage, could prove to be more successful in future X-cell transmission studies. Furthermore, using fish with advanced pseudotumours, close to rupture, could be important for successful transmission to occur between experimental fish. However, both gadoid and pleuronectid fish with X-cell pseudotumours have very poor survival rates after capture [3,22] which may hinder such long-term cohabitation experiments. In addition, lesion regression has also been observed in captive X-cell infected dab [22], suggesting that environmental conditions, such as temperature, may also be critical for the experimental transmission of X-cell parasites to take place and for pseudotumours to develop.

### Geographical and environmental niches

X-cell pseudotumours in numerous wild fish populations have been reported to occur within extremely narrow geographic locations [4,23], being found in high numbers at one location and almost absent in adjacent localities or bays. This irregular distribution pattern originally led to suggestions that the X-cell condition was caused by localised coastal pollution [24,25]. A comprehensive study by Katsura et al. [4] of the distribution of X-cell infected fish in Hokkaido, however, revealed that the presence of X-cell pseudotumours was not related to coastal pollution, but had a strong correlation to the substrate type and amount of tidal water exchange. They found a very low frequency of infected fish (0.02-0.27%) on the north side of the Notsuke peninsula where a strong tide flows from north to south, but south of the peninsula, where the bay is protected from the strong current and the waters are 'stagnated and the bottom quite muddy', fish had a 12.3% prevalence of X-cell pseudotumours. Similar findings for the distribution patterns of populations of X-cell infected fish in Europe [23] suggest that certain environmental conditions such as substrate type and tidal flow potentially favour the survival and development of a free-living X-cell stage or can maintain an obligate alternate host that has a restricted zone for habitation.

### Species specificity

Katsura et al. [4] suggested that species specificity was present among X-cell infections in flatfish from Hokkaido, as some pleuronectid fish (*Liopsetta pinnifasciata *and *Limanda punctatissimus*) were collected in large numbers from numerous sites with muddy substrates with other X-cell infected pleuronectid fish being present, but they were never found to be infected with X-cells themselves. During this study we have shown that the same parasite causes X-cell pseudotumours in both *H. dubius *and *P. obscurus *from geographically distant locations in northern Japan, demonstrating that X-cell parasites are not species specific in some pleuronectid fish. It would be interesting to expand on this study and investigate whether the same parasite infects all seven pleuronectid fish known to be susceptible to epidermal X-cells infections in northern Japan. In our experimental transmission study, we used two fish species due to their availability from hatcheries; *Verasper moseri *(family Pleuronectidae) and *Paralichthys olivaceus *(family Paralichthyidae). *Verasper moseri*, sampled from the same location we sampled *P. obscurus *for this study, have been shown to be susceptible to infection with X-cell parasites causing characteristic epidermal pseudotumours [4], however it remains possible that the X-cells from *P. obscurus *are a different species to those in *V. moseri*. The Japanese flounder, *P. olivaceus*, has not previously been reported to be susceptible to infection with X-cell parasites and was included in this study due to its availability from hatcheries and its economic importance. We believe that *P. olivaceus *is probably not susceptible to the X-cell parasite infecting other flatfish species in northern Japan, as no reports are found in the literature and they are not pleuronectid fish. Its inclusion in this experiment could also have been valuable as a negative control had transmission been successful for *V. moseri*. In the gadoid transmission experiment we were able to use the same species for both donor and experimental fish, hence eliminating any species specificity concerns for the experiment.

### Phylogenetic relationships

Using SSU rDNA alone, Miwa et al. [2,5] were able to demonstrate that X-cell parasites infecting different fish hosts (goby and flathead flounder) were clearly related, but were not able to demonstrate a stable phylogenetic relationship between the X-cell group and the other taxa used in their analyses. In a more comprehensive phylogenetic analysis, again limited to SSU rDNA, Freeman [1] confirmed monophyly for the X-cell group, but could only place the X-cell clade within the superphylum Alveolata and suggested that they were possibly related to both apicomplexans and dinoflagellates, and referred to them as basal myzozoans. The present study is the first to include a SSU rDNA sequence for the cod X-cell parasite, and our phylogenetic analyses confirm monophyly for the X-cell group, but again fail to locate the X-cell clade convincingly within any of the recognised alveolate groupings. However, the inclusion of the sequence from cod X-cells has altered the expected topology of the tree for the alveolate group. *Parvilucifera *spp. that normally group with other perkinsids at the base of the dinoflagellates clade now occupy an unresolved branch in the tree. It is evident from recent molecular studies [[1,2,5], present study] that SSU rDNA sequence data alone is not sufficiently informative to robustly place the X-cell group in phylogenetic studies and that additional more conserved gene regions should be studied to clarify their phylogenetic relationship with other alveolate taxa.

### Suggested life cycle for X-cell parasites

To date, numerous histopathological and ultrastructural studies of X-cell parasites in fish have failed to reveal a developmental stage beyond the familiar X-cell, either locked in host tissue or as a free form in advanced pseudotumours. Here, we demonstrate that non-amoeboid X-cells are contained within discrete sac-like structures that are loosely attached to the fish host and that the X-cells from these sacs are able to tolerate seawater conditions. We believe that this represents good evidence that detachment from fish occurs naturally. Sac-like structures have not been observed or reported from pseudobranchial pseudotumours in gadoids or in X-cell gill lesions. However, free-floating X-cells have been observed in advanced pseudobranchial pseudotumours in Atlantic cod [18] and ruptured pseudotumours have been reported from X-cell gill lesions [19], which may serve as alternative release mechanisms for non-epidermal X-cells.

**Figure 7:**
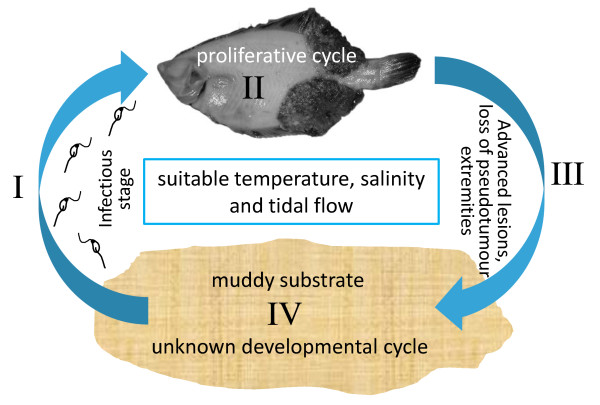
**Proposed life cycle for the X-cell parasite from flatfish in northern Japan**. The proposed life cycle consists of four stages. (I) An infective stage, either flagellated or equipped with suitable apparatus for host penetration/infection, which is released into the environment to infect a fish host. (II) A proliferative cycle starts in the fish which develops into large epidermal pseudotumours. (III) As the pseudotumours mature, sac-like extremities are lost from the host fish and fall to the seabed where the X-cells are released into the substrate. (IV) In the substrate, either a free-living development occurs or the X-cells find an alternate host where development proceeds.

We do not believe that fish represent a dead-end or incidental host for X-cells. X-cells have evolved very specific tissue tropisms in the fish species they infect and have been reported from 5 teleost orders globally [1], suggesting a long-term and well-established host parasite relationship. Therefore, we can conclude that X-cell development in the fish host is purely a proliferative phase that leads to further development either outside of the fish host or in an alternate host. The lack of direct transmission for the X-cell parasites in this study supports this theory. Figure 7 shows four proposed stages in the life cycle for the X-cell parasite infecting flatfish in northern Japan.

I. The infective stage to new fish hosts is either flagellated or equipped with suitable apparatus for host penetration and is released into the environment either from an alternate host or from a stage that developed during a free-living environmental phase in the life cycle.

II. A proliferative cycle starts in the fish which develops into large epidermal pseudotumours, each containing large quantities of X-cells.

III. As the pseudotumours mature, discrete sac-like structures are lost from the host fish and fall to the seabed where the X-cells are released into the substrate.

IV. If the substrate type is suitable, either free-living environmental development occurs or the X-cells infect and develop within an alternate host to produce infective stages for new fish hosts.

Understanding the life cycle of parasites that can infect commercially valuable fish species is important. In Europe, cod farming is an emerging industry and is still in the relatively early stages of development. However, pseudobranchial X-cell infections in Atlantic cod have already been shown to cause serious pathology and mortalities in farmed fish [26]. Identifying farm-sites that might favour the propagation of or allow X-cells parasites to become established in surrounding sediments will assist future aquaculture endeavours, such as gadoid farming.

## Conclusions

The presence of discrete sac-like structures, filled with non-amoeboid X-cells, which are readily detached from the host fish and the lack of fish to fish transmission for both parasites in this study, suggests that other stages exist in the X-cell life cycle. This type of developmental cycle for X-cells may explain the lack of typical alveolate features seen in fish X-cells, which may be present in other stages of the life cycle. We suggest that X-cells have a second development phase either as free-living environmental organisms in the marine sediments or in an alternate host.

Analysis of SSU rDNA from X-cells infecting flatfish in Japan confirmed that the same X-cell parasite can infect more than one species of fish. Phylogenetic analyses of X-cell SSU rDNA places them as a monophyletic group in the alveolates. PCR analysis of infected flatfish showed that X-cell DNA is detectable in non-pseudotumour tissues, suggesting that some X-cells are motile in fish and may be responsible for autoinfection in the fish host.

## Competing interests

The authors declare that they have no competing interests.

## Authors' contributions

ME and MAF collected material in Iceland and set up the transmission experiment, ME maintained the experiment for the duration. MY and KW organised sample collection in Japan, KO and MF set up the transmission experiment and KM maintained the experiment for the duration. MAF performed wax histology and DNA analyses, developed the nested PCR and drafted the manuscript. APS cut and viewed semi-thin plastic sections of the sac-like structures.

## Supplementary Material

Additional file 1 **Supplementary data**. Additional small subunit ribosomal DNA sequences used in the phylogenetic analyses. Click here for file
