# Supplementary material for: Molecular identification and transmission studies of X-cell parasites from Atlantic cod Gadus morhua (Gadiformes: Gadidae) and the northern black flounder Pseudopleuronectes obscurus (Pleuronectiformes: Pleuronectidae)
Source: Parasit Vectors. 2011 Feb 8;4:15. doi: 10.1186/1756-3305-4-15 (PMC3045979; doi:10.1186/1756-3305-4-15)
Supplement: Additional file 1 — Supplementary data. Additional small subunit ribosomal DNA sequences used in the phylogenetic analyses. [file 1756-3305-4-15-S1.PDF]

Supplementary data: additional small subunit ribosomal DNA sequences used in the phylogenetic analyses

Group / Organism	Accession No	Group / Organism	Assession No
**X-cells**		**Cercozoa**	
L. limanda (Scotland)	EU878172	*Euglypha acanthophora*	AJ418788
H. dubius (Japan)	AB112470	*Massisteria marina*	AF174374
A. flavimanus (Japan)	AB451874	*Heteromita globosa*	U42447
		Cercomonas sp.	U42451
**Alveolata; Perkinsea**			
Perkinsus marinus	X75762	**Cercozoa; Haplosporidia**	
Perkinsus mediterraneus	AY517647	*Haplosporidium nelsoni*	X74131
Parvilucifera infectans	AF133909	*Bonamia ostreae*	AF262995
Parvilucifera sinerae	EU502912	*Minchinia teredinis*	U20320
		Urosporidium crescens	U47852
**Alveolata; Dinophyceae**			
Hematodinium sp.	AF286023	**Stramenopiles**	
Dinophyceae sp.	AM408889	*Cyclonexis annularis*	AF123292
Cochlodinium fulvescens	AB288381	*Lagynion scherffelii*	AF123288
Paulsenella vonstoschii	AJ968729	*Blastocystis hominis*	AY244621
Pfiesteria sp.	AF218805		
		**Excavata; Heterolobosea**	
**Alveolata; Apicomplexa**		*Naegleria gruberi*	M18732
Theileria sp.	AY421708	*Vahlkampfia lobospinosa*	M98052
Babesia sp.	AB053216		
Toxoplasma gondii	U03070	**Excavata; Parabasalidea**	
Cryptosporidium parvum	AY204241	*Tritrichomonas foetus*	AY055799
Cryptosporidium wrairi	AF115378	*Pentatrichomonas hominis*	AF124609
Gregarina polymorpha	AF457129		
Gregarina niphandrodes	AF129882	**Lobosea; Euamoebida**	
Eimeria tenella	DQ136181	*Hartmannella abertawensis*	DQ190241
Adelina grylli	DQ096836	*Glaeseria mira*	AY294146
Ascogregarina taiwanensis	DQ462454		
Leidyana migrator	AF457130	**Ichthyosporea**	
		Dermocystidium sp.	AF533950
**Alveolata; Ciliophora**		*Ichthyophonus hoferi*	U25637
Colpoda inflata	M97908	*Psorospermium haeckelii*	U33180
Oxytricha nova	X03948		
Paramecium tetraurelia	X03772	**Fungi**	
		Saccharomyces cerevisiae	AF548094
